# Identification of Pilots’ Fatigue Status Based on Electrocardiogram Signals

**DOI:** 10.3390/s21093003

**Published:** 2021-04-25

**Authors:** Ting Pan, Haibo Wang, Haiqing Si, Yao Li, Lei Shang

**Affiliations:** College of General Aviation and Flight, Nanjing University of Aeronautics and Astronautics, Nanjing 211106, China; nuaapanting@nuaa.edu.cn (T.P.); nhwanghaibo@nuaa.edu.cn (H.W.); liyao1224@nuaa.edu.cn (Y.L.); shanglei@nuaa.edu.cn (L.S.)

**Keywords:** fight safety, pilot fatigue, electrocardiogram, feature analysis, identification of fatigue

## Abstract

Fatigue is an important factor affecting modern flight safety. It can easily lead to a decline in pilots’ operational ability, misjudgments, and flight illusions. Moreover, it can even trigger serious flight accidents. In this paper, a wearable wireless physiological device was used to obtain pilots’ electrocardiogram (ECG) data in a simulated flight experiment, and 1440 effective samples were determined. The Friedman test was adopted to select the characteristic indexes that reflect the fatigue state of the pilot from the time domain, frequency domain, and non-linear characteristics of the effective samples. Furthermore, the variation rules of the characteristic indexes were analyzed. Principal component analysis (PCA) was utilized to extract the features of the selected feature indexes, and the feature parameter set representing the fatigue state of the pilot was established. For the study on pilots’ fatigue state identification, the feature parameter set was used as the input of the learning vector quantization (LVQ) algorithm to train the pilots’ fatigue state identification model. Results show that the recognition accuracy of the LVQ model reached 81.94%, which is 12.84% and 9.02% higher than that of traditional back propagation neural network (BPNN) and support vector machine (SVM) model, respectively. The identification model based on the LVQ established in this paper is suitable for identifying pilots’ fatigue states. This is of great practical significance to reduce flight accidents caused by pilot fatigue, thus providing a theoretical foundation for pilot fatigue risk management and the development of intelligent aircraft autopilot systems.

## 1. Introduction

With the rapid development of the air transport industry in aircraft design, manufacturing, maintenance, and other aspects, the reliability and safety of aircraft have been significantly improved. However, the proportion of flight accidents caused by human factors in the overall number of accidents has not decreased [1,2,3]; these are especially caused by pilot fatigue [4,5]. The noise and vibration of the cabin, air pressure changes, long-haul flights, high-load work, circadian rhythm disturbance, and lack of sleep lead pilots to often be in a state of fatigue [6]. Fatigue could easily lead to problems such as reduction in operational ability, misjudgment, flight illusions, etc., and even cause serious flight accidents. Therefore, how to identify the real-time fatigue state of pilots quickly and accurately has become a core scientific problem that needs to be solved urgently in the field of aviation safety. The identification of pilot fatigue is of great theoretical and practical significance for achieving pilot fatigue risk control, health management, and real-time safety warning for autopilot systems.

When pilots are tired, their heart function, nerve function, respiratory function, and other related functions change accordingly. Therefore, the fatigue status could be reflected by electroencephalogram (EEG), electrocardiogram (ECG), electromyography (EMG), or other physiological indicators [7,8,9,10,11,12]. EEG and ECG are widely studied for their good indication of fatigue [13,14]. EEG has been known as the “gold indicator” of monitoring fatigue and is widely used in fatigue detection [15,16,17,18]. However, in the process of detecting pilot fatigue, EEG signal acquisition is can be susceptible to interference by external factors: it has high requirements for the data acquisition environment, is invasive to pilots (for example, electrodes need to be attached to the participant’s head), and a low signal-to-noise ratio; thus, it is difficult to carry out in dynamic simulations or actual flight experiments. ECGs reflect the activity of the heart. There is a correlation between heart rhythm and autonomic nervous system (ANS) [19]. The autonomic nervous system is composed of sympathetic nerves (SNS) and parasympathetic nerves (PSNs), which regulate the arousal level of the human body. In the process of fatigue, the activity of sympathetic nerves and parasympathetic nerves change significantly. Thus, the fatigue state of the human can be reflected to a certain extent by the ECG signal [20,21,22,23].

Some scholars have conducted research on ECG indicators that reflect pilot fatigue. Fan et al. selected 30 military cadets to conduct airfield traffic pattern simulation experiments for four consecutive hours to collect the ECG signals of the participants. The analysis results show that there are statistical differences in heart rate and its variability time domain index RMSSD before and after the flight mission [24], which could be used to quantitatively analyze pilots’ fatigue status. Hanakova et al. collected the ECG signals of eight flight cadets through flight simulation experiments (eight simulated IFR flights within 24 h) and analyzed them in the way of time domain, frequency-domain, and Poincaré plot. The study found that the Poincaré plot of the ECG data could also reflect the fatigue state of the pilots [18].

Some studies on the fatigue state identification method based on the ECG index have an important reference significance for the identification of pilots’ fatigue state. Patel et al. analyzed the ECG data, extracted the heart rate variability as a physiological index, and used a neural network to identify the fatigue state caused by long-time driving [25]. Sang-Joong et al. collected the ECG signal from the driver’s palm throughout a two-hour driving experiment, extracted the time domain and frequency domain indicators, and used the autonomic nervous system balance graph to identify the drivers’ drowsiness and fatigue [26]. Based on data of drivers’ ECG, Bhardwaj et al. selected the time domain, frequency domain, and Poincaré scatter diagram indicators and used the deep auto-encoder classifier to classify the non-fatigue and fatigue states [17]. Xu et al. selected the time domain and frequency domain characteristic indexes from the electrical signal of the Drozy database center as the model input, adopted a random forest classifier and support vector machine (SVM) to establish the fatigue state classification model, and found that the random forest classifier has a higher accuracy in identifying driving fatigue [27]. Li et al. used a wavelet transform to extract heart rate variability features of the ECG signal and adopted SVM as a classifier to identify fatigue state [28]. Munla et al. extracted and preprocessed the drivers’ ECG signal and analyzed the drivers’ heart rate variability. Several parameters in the heart rate variability signal were selected. The SVM based on the radial basis function was used as a classifier to identify the drivers’ mental activity state [29].

These results show that the time domain, frequency domain, and non-linear indexes extracted from the ECG signals could quantitatively measure the fatigue state of pilots, and the fatigue state could be identified based on the classification model. Thus, this provides a strong theoretical basis and technical support for the study of pilot fatigue state identification. However, there is little research on fatigue in civil aviation at home and abroad, and there still exist many problems to be solved. In previous studies, the participants of pilots’ fatigue state identification generally used ordinary people such as college students and graduate students; unfortunately, only a few of the participants were pilots. To become a pilot, we need to go through a series of strict selection, training, and assessments and obtain the relevant certificates. Compared with other people, pilots may show different physical and psychological states during flight missions. In some previous studies, computer simulation experiments have been used. This is somewhat different from the real flight environment and flight mission. Compared with the computer simulation experiment, the environment provided by the flight simulator is closer to the real flight environment. In some previous studies, the time window for feature extraction of ECG signals characterizing pilots’ fatigue state is generally not less than 5 min. If the time window is shortened, it may be more conducive to the quick identification of pilots’ fatigue state.

In this paper, to meet the requirements of pilots’ natural driving and safe flight, ECG signals with the advantages of a high sampling rate, reliable data, easy operation, and non-intrusive nature are selected to identify the fatigue state of pilots in combination with subjective self-assessment, thus guaranteeing the real-time performance and accuracy of pilot fatigue data. Firstly, the flight simulation experiment is carried out by using a Cessna 172 flight simulator. The fatigue state of pilots is defined by the Samn–Perelli 7-Level fatigue scale, and the ECG data of pilots under different fatigue states are obtained. Secondly, the ECG signal of pilots is denoised by information processing techniques such as wavelet threshold transform. The time domain, frequency domain, and non-linear indexes of the ECG signals are extracted with a time window of 100 s. Thirdly, the feature vectors of these indexes are extracted by way of the Friedman test and principal component analysis (PCA). The change in trend of the ECG characteristic indexes in different fatigue states is analyzed. Finally, the learning vector quantization (LVQ) is used to train and classify the characteristic vectors of pilot fatigue so as to realize the identification of the pilots’ fatigue state.

The rest of this article is organized as follows. The research methods are introduced in Section 2. Details of the experimental contents are described in Section 3. The results will be analyzed and discussed in Section 4. Section 5 presents the conclusion.

## 2. Method

**Figure 1:**
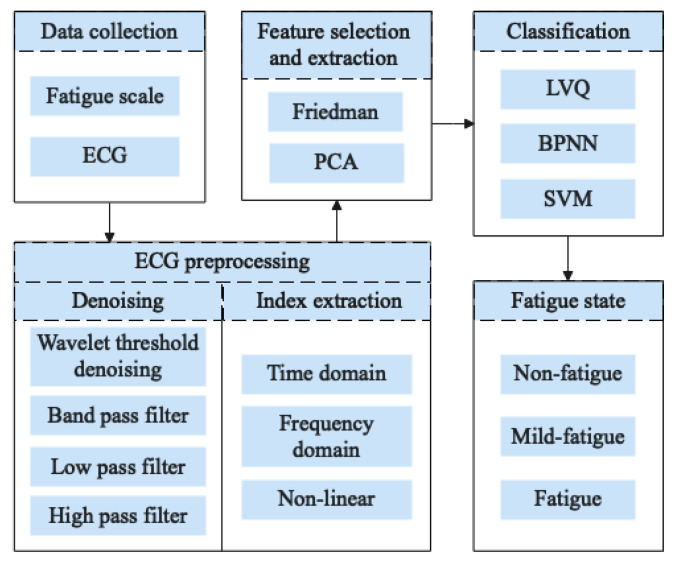
Research architecture diagram.

The research framework of this paper is displayed in Figure 1. To identify the fatigue state of pilots, ECG data and the Samn–Perelli 7-Level fatigue scale data are collected through flight simulation experiments. The obtained ECG data are preprocessed. Then, time domain, frequency domain, and the non-linear characteristic indexes of the ECG data are selected and extracted by the Friedman test and PCA. Based on the results of feature selection and extraction, the pilots’ fatigue state identification model is established by using the LVQ neural network. The pilot’s non-fatigue, mild fatigue, and fatigue states are identified. The identification results are compared with those of BPNN, SVM and other traditional methods.

### 2.1. Data Preprocessing

#### 2.1.1. Denoising of ECG Signals

In general, the collected ECG signals have certain noise, such as white noise, motion artifacts, power line interference, EMG interference, and baseline drift [30]. In this paper, band pass filtering, low pass filtering, and high pass filtering are adopted to remove power line interference, EMG interference, and baseline drift noise in the ECG signals, respectively. Additionally, wavelet threshold denoising is used to remove white noise and motion artifacts, as shown in Table 1.

The ECG signals are collected by a wireless ECG sensor in this paper. The frequency range of an ECG signal is 0.01–200 Hz. The frequency range of EMG interference is 5–2000 Hz. The frequency range of baseline drift is 0.1–0.5 Hz. The frequency of power line interference is 50 Hz. A low pass filter is utilized to remove high-frequency noise greater than 100 Hz, which can eliminate most EMG interference. Correspondingly, a high pass filter is used to remove signals with a frequency below 0.5 Hz to eliminate the effect of baseline drift. Electromagnetic interference in the surrounding environment may also cause power line interference [31]. At present, the researchers still consider power line interference when processing data with wearable ECG acquisition devices [32,33]. Accordingly, the 50 Hz band filter is used to remove the power line interference.

**Figure 2:**
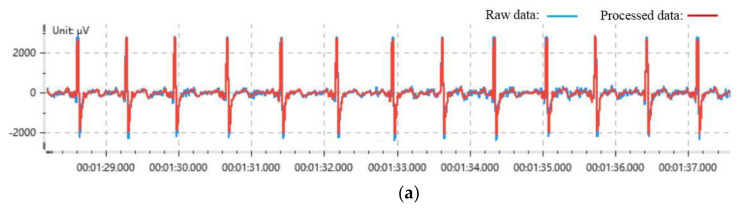

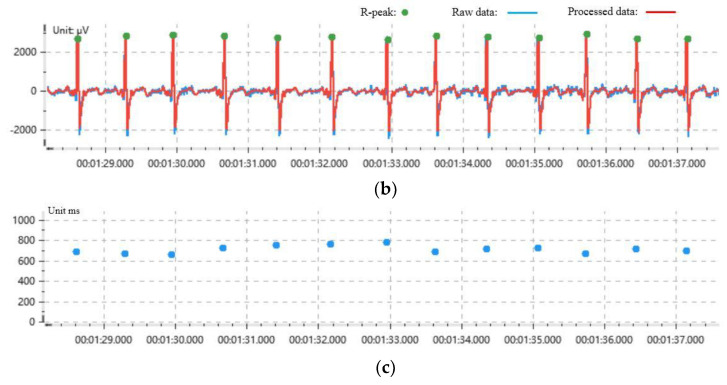
ECG Preprocessing. (**a**) ECG signal data before and after denoising. (**b**) The results of R-peaks extraction. (**c**) The results of RR intervals.

Wavelet threshold denoising can remove white noise [34]. We chose Daubechies-4 as the wavelet generating function, and the threshold selection is shown in Equation (1) [35]. After the ECG signal is decomposed by the wavelet threshold denoising, the wavelet coefficient of the effective signal is larger and that of the white noise is smaller [36]. Thus, we can remove the white noise. At the same time, motion artifacts can also be removed by wavelet threshold denoising [37,38]. The comparison of some ECG signal data before and after denoising is exhibited in Figure 2a.

(1) $$T=\sqrt{2*\log\left(N\right)}$$

where N represents the length of the signal.

#### 2.1.2. ECG Index Extraction

On the basis of denoising the ECG signal, the peak value of the R peaks of QRS is extracted, and the RR intervals are further obtained. At present, a differential threshold method is the most commonly used method for detecting the ECG signal waveform, which is a combination of the threshold method and differential operation. The basic idea of detection is as follows: first of all, Equation (2) is used to carry out the first order difference to find all the inflection points of the ECG signal. Secondly, using Equation (3), the second order difference of the ECG signal is performed to find all peaks of the ECG signal. Finally, Equation (4) is used to determine the peak threshold and find the peak point of the R wave. Then, we can obtain the RR interval. The results of R-peaks extraction and RR intervals are shown in Figure 2b,c.

(2) $$f^{\prime }\left(x_{k}\right)=\frac{f\left(x_{k+1}\right)-f\left(x_{k}\right)}{x_{k+1}-x_{k}}$$

(3) $$f^{\prime \prime }\left(x_{k}\right)=\frac{f^{\prime }\left(x_{k+1}\right)-f^{\prime }\left(x_{k}\right)}{x_{k+1}-x_{k}}$$

(4) Threshod=(max(x)−min(x))∗0.7+min(x)

where Threshod is the fixed threshold that needs to be set, max(x) is the maximum value of the ECG signal in the detected time window, min(x) is the minimum value of the ECG signal in the detected time window, and 0.7 is the empirical value.

Some meaningful indexes representing the ECG signal are extracted from the time domain, frequency domain, and non-linear indexes. These indexes are listed in Table 2, including eight time domains, eight frequency domains, and five non-linear ones.

### 2.2. Feature Selection and Extraction

#### 2.2.1. Friedman Test

In order to test the normality of the indexes, the Kolmogorov–Smirnov test is adopted. All the indexes do not conform to the normal distribution. As a result, a non-parametric test method, namely, the Friedman test, is adopted. The Friedman test is a method that uses rank to judge whether there are significant differences in multiple overall distributions [39]. We use the Friedman test to select some indicators that have significant correlation in non-fatigue, mild fatigue, and fatigue states.

Suppose that the null hypothesis is $H_{0}$, and the alternative hypothesis is $H_{1}$. $H_{0}$ represents that multiple samples come from populations with no significant difference in size. $H_{1}$ represents ones with significant difference in size. The test value is calculated by Equation (5):

(5) $$X^{2}=\frac{12}{nm(m+1)}\sum R_{j}^{2}-3n(m+1)$$

where m and n are the number of groups and the sample size in the group, respectively, and $R_{j}$ represents the rank sum of column.

According to the significance level (α) and the degree of freedom (m−1) determined in advance, the corresponding critical value ($F_{\alpha [m-1]}^{2}$) is obtained from the Chi-square table. If $X^{2}<F_{\alpha [m-1]}^{2}$, the null hypothesis is correct. Otherwise, the alternative hypothesis is correct.

#### 2.2.2. Principal Component Analysis

After feature selection based on the Friedman test, there may be a certain correlation between the characteristic indexes. There will be data coupling. Principal component analysis (PCA) is adopted to further eliminate the mutual effect among feature extraction to reduce the complexity. Principal component analysis is a statistical method of dimensionality reduction. Many indexes with a certain correlation are recombined, and the important dimensions of them are extracted. A new set of independent comprehensive indexes are utilized to replace the original indexes. Thus, redundant information is eliminated.

The number of principal components is selected according to the principle that the cumulative contribution rate of principal components reaches 85%. Principal component contribution rate $W_{d}$ and cumulative contribution rate $s_{f}$ are calculated by Equations (6) and (7). The value of factor load is calculated by Equation (8).

(6) $$W_{d}=\frac{\lambda_{d}}{\sum_{k=1}^{p}\lambda_{k}},\;(d=1,2,\cdots ,p)$$

(7) $$s_{f}=\frac{\sum_{k=1}^{f}\lambda_{k}}{\sum_{k=1}^{p}\lambda_{k}},\;(f=1,2,\cdots ,p)$$

where $\lambda_{d}$ represents the eigenvalue of the covariance matrix, and p represents the pth feature of the sample.

(8) $$l_{kd}=\sqrt{\lambda_{k}}u_{kd},\;(k,d=1,2,\cdots ,p)$$

where $u_{kd}$ represents the kth element of the orthogonalized eigenvector.

### 2.3. Learning Vector Quantization

**Figure 3:**
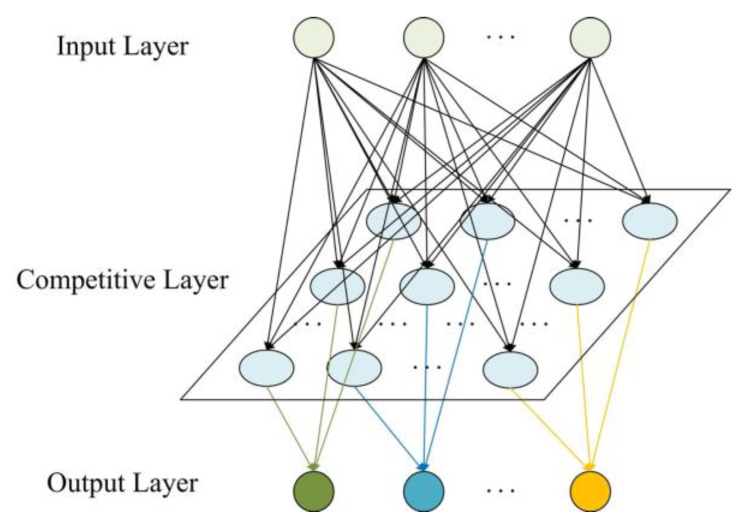
LVQ neural network.

Learning vector quantization is a kind of forward and supervised neural network evolved from a competitive algorithm by Finnish scholar Kohonen [40]. The LVQ neural network consists of an input layer, competition layer, and output layer, as shown in Figure 3. The classification results can be obtained only through the internal interaction of the input layer, competitive layer, and output layer [41,42]. The LVQ neural network has better recognition and convergence characteristics for complex and scattered feature datasets.

**Figure 4:**
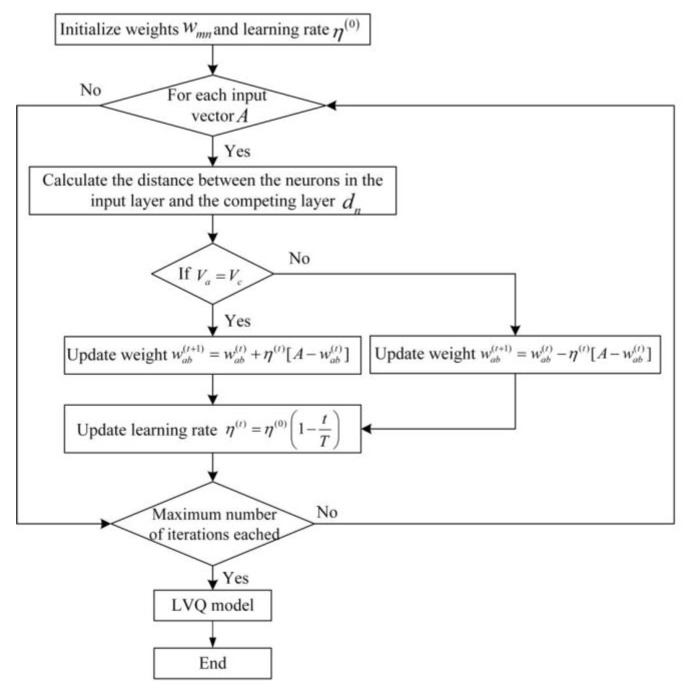
The training steps of LVQ.

As shown in Figure 3, the connection mode between the input layer and competition layer is a full connection. The connection mode between the competition layer and linear output layer is a partial connection. When a vector is input into the network, the neuron in the competition layer closest to the input vector is activated to win the competition. At this time, the state is “1”, while the rest of the neurons in the competition layer are all “0”. The training steps of the LVQ are as show in Figure 4.

## 3. Materials

### 3.1. Participants

The sample size of the experiment was 30 pilots who had obtained a Private Pilot License, Commercial Pilot License, and Instrument Rating License. The basic information of the participants is described in Table 3. Based on the existing research of fatigue state based on the ECG signals, a small sample of ECG signals can also reflect certain regularity [17,43]. The more experimental samples there are, the more accurate the experimental data are. However, the experiment is costly and time-consuming. It is difficult to analyze when the amount of data is large. Based on the above factors, the number of pilots selected in this article is 30. The selected pilots are all right-handed. Their vision or corrected vision is normal, and there is no serious history of tumor, nephritis, endocrine disorders, etc. Before the experiment, the participants were required to ensure adequate sleep and avoid strenuous exercise. In addition, 24 h before the experiment, they were asked to avoid smoking, drinking, and other behaviors that excite or inhibit the central nervous system so that the participants could maintain a good mental state.

The experimental process is strictly in accordance with the Helsinki Declaration. All experimental personnel received a complete explanation of the experimental procedures and equipment. All the participants were familiar with the whole experimental process. They volunteered to participate in the experiment and filled in a written consent form.

### 3.2. Flight Simulation Experiment

#### 3.2.1. Subjective Self-Evaluation

The Samn–Perelli 7-Level fatigue scale is a subjective method for evaluating fatigue state. The contents of the Samn–Perelli 7-Level fatigue scale are listed in Table 4. In this paper, the Samn–Perelli 7-Level fatigue scale is utilized to evaluate the fatigue status of the pilots. The fatigue level is divided into 7 levels. Each fatigue level corresponds to a different score. A score of 1 represents very alert and fully awake. A score of 7 indicates exhausted. The pilot chooses the closest fatigue level according to his own fatigue state. Generally, a fatigue scale score of no more than 3 is defined as non-fatigue status, a score greater than 3 but no greater than 5 is defined as mild fatigue status, and a score greater than 5 is defined as fatigue status [44]. The experiment was conducted at the following times: 9:00–11:00, 14:00–16:00, and 19:00–21:00. The participants filled in the Samn–Perelli 7-Level fatigue scale before and after the flight mission in each time period and obtained their own fatigue scale score. Fatigue level depends on the subjective feelings of the participants, and it is easily affected by many factors, such as individual differences, emotions and, environment. It may be different from the actual fatigue level. Using the experimenter’s assessment of the fatigue level of the participants can improve the accuracy of the fatigue level [45]. While the participants filled in the scale, the experimenters also filled in the Samn–Perelli 7-Level scale according to the fatigue state of the pilot. The average value is taken as the final score of the pilot’s fatigue evaluation, which is calculated based on the fatigue scale filled in by the participants and experimenters.

#### 3.2.2. Experimental Equipment

**Figure 5:**
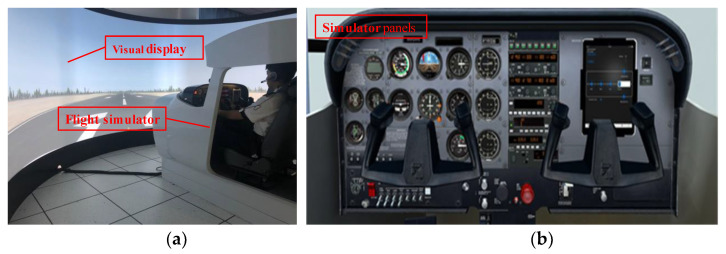

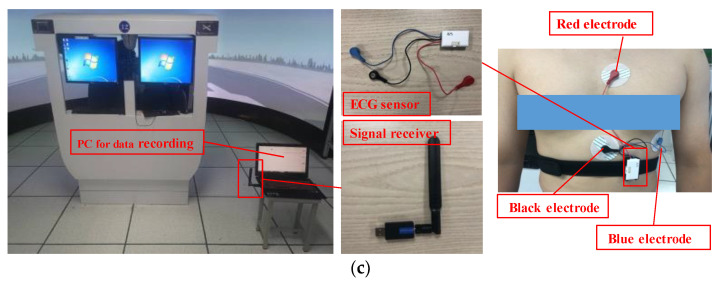
Flight simulation experiment equipment. (**a**) Cessna 172 flight simulator. (**b**) Simulator panels of Cessna 172 flight simulator. (**c**) The wireless wearable ECG data acquisition and recording device.

The Cessna 172 flight simulator which is manufactured by Tianjin ZTXY Aviation Technology Co., Ltd., Tianjin (China), is utilized for flight simulation experiment, as shown in Figure 5a,b. The airport, weather environment, training mode, etc. could be set in the flight simulator. The flight simulator has a good simulation effect and immersion. Pilots wore wireless wearable ECG sensors to perform flight missions in the Cessna 172 flight simulator. The wireless wearable ECG data acquisition and recording device are depicted in Figure 5c, and its parameters are given in Table 5. The ECG sensor is produced by KINGFAR Co., Ltd., Beijing (China). The ECG sensor is worn on the chest of the participants; the specific location is shown in Figure 5c.

#### 3.2.3. Experimental Task

**Figure 6:**
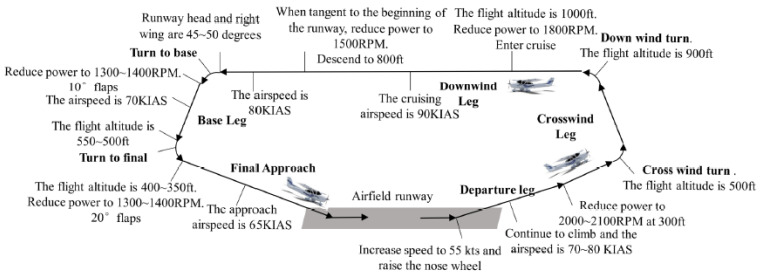
Schematic diagram of the airfield traffic pattern mission process.

**Figure 7:**
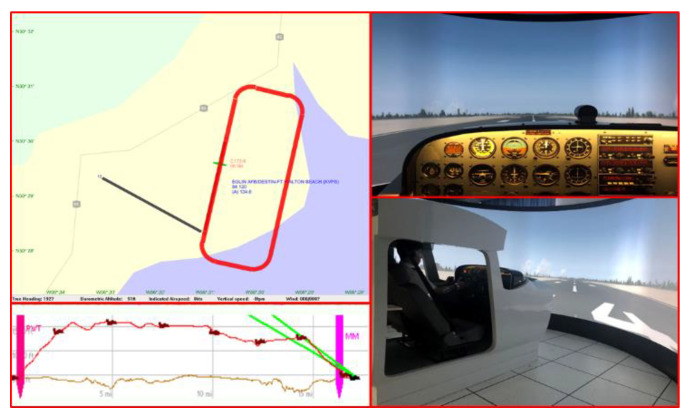
Schematic diagram of the pilot completing the airfield traffic pattern.

The visual manual airfield traffic pattern mission operation includes the basic flight operations during flight, such as rolling, take-off, climbing, turning, descent, landing, etc. Here, the visual manual airfield traffic pattern mission is used as the experimental content. The process of the visual manual airfield traffic pattern is displayed in Figure 6. The pilots continuously performed the flight mission during the experimental period. During the entire simulation flight process, the clear and windless Eglin Air Force Base (AFB) was selected. The simulated airfield traffic pattern process completed by the pilots at the Eglin Air Force Base is indicated in Figure 7.

#### 3.2.4. Experimental Process

**Figure 8:**
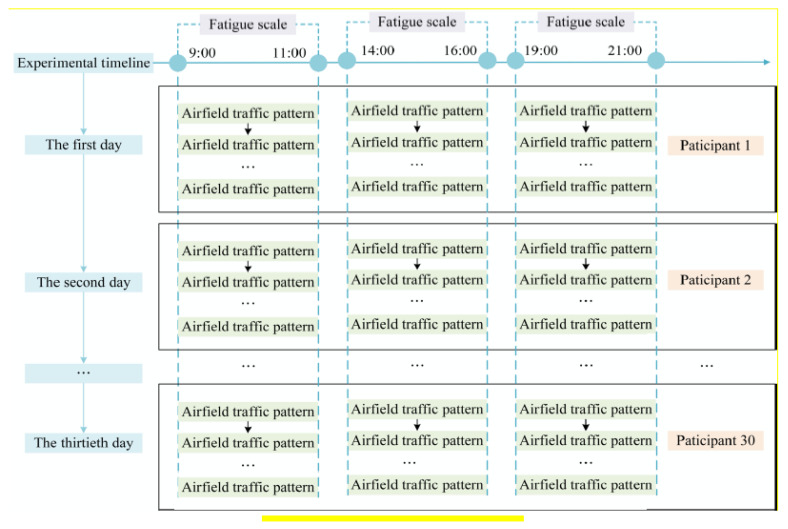
Experimental process.

On the day before the experiment, the participants rested before 23:00 to ensure that they had 8 h of adequate sleep. In addition, they participated in the experiment from 9:00 to 11:00, 14:00 to 16:00, and 19:00 to 21:00 on the day of the experiment. Moreover, they were deprived of their right to sleep at noon on the experimental day. The experiment lasted for 30 days. One pilot participated in the experiment every day. The ECG data of the pilots were collected at 9:00–11:00, 14:00–16:00, and 19:00–21:00. This is about six hours a day. The experimental process is illustrated in Figure 8.

### 3.3. Data Collection

In this paper, the ECG data of 30 pilots were obtained through flight simulation experiments. In order to eliminate the effect of the beginning and the end of the experiment on the pilots’ psychology, the ECG data from 9:45 to 10:15, 14:45 to 15:15, and 19:45 to 20:15 were selected for analysis. Based on the study of driver fatigue by using a 100s time window of the ECG, the 100 s time window was selected to intercept the ECG data [46]. A total of 1440 effective samples were extracted, and the sample size of each time period was 480. ErgoLAB software which is produced by KINGFAR Co., Ltd., Beijing (China), is used to calculate the indexes of the samples. The calculated indexes include time domain, frequency domain, and nonlinearity indexes, and the specific indexes are shown in Table 2. Some experimental sample data are presented in Table 6.

## 4. Results and Discussion

### 4.1. Fatigue Scale Analysis

**Figure 9:**
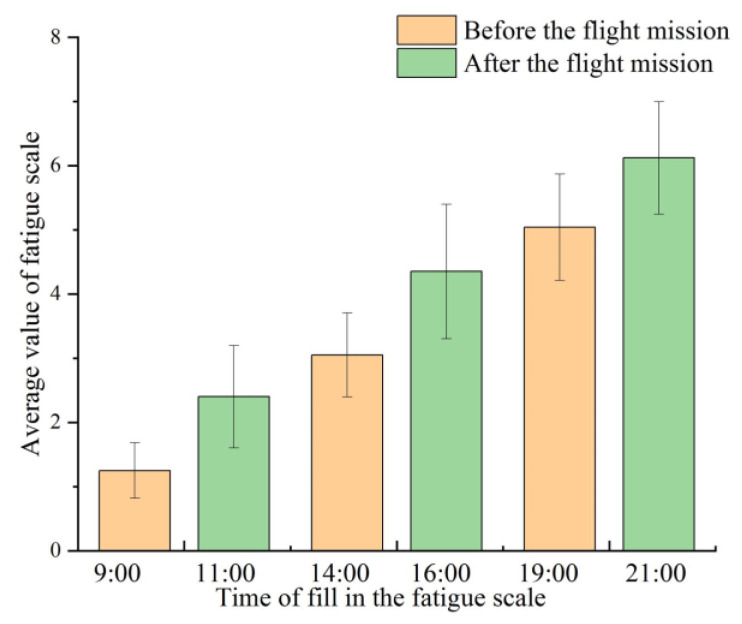
The statistics of Samn–Perelli 7-Level fatigue scale.

Figure 9 shows the statistics of the Samn–Perelli 7-Level fatigue scale. It can be seen from Figure 9 that the pilots’ fatigue levels are significantly different in the three time periods of 9:00–11:00, 14:00–16:00, and 19:00–21:00. At 7:00 and 9:00, the averages of the fatigue scale are less than 3. At 14:00 and 16:00, the averages of the fatigue scale are greater than or equal to 3 but no greater than 5. At 19:00 and 21:00, the averages of the fatigue scale are greater than or equal to 5. Their fatigue scores in the three time periods will show an increasing trend with the passage of time. Thus, the pilot fatigue states corresponding to these three time periods could be divided into non-fatigue, mild fatigue, and fatigue. Non-fatigue, mild fatigue, and fatigue state are used as the labels for model training.

### 4.2. Feature Selection and Extraction of Fatigue State

We randomly selected 22 participants and used the data of these 22 participants as the training set. The data from the remaining 8 participants were used as the test set. The training set contains 1152 samples, and the sample size of each time period in the training set is 384. The test set contains 288 samples, and the sample size of each time period in the test set is 96. The proportion of non-fatigue, mild fatigue, and fatigue samples in the training set and test set is 1:1:1. We selected and extracted the features from the training set to establish the pilots’ fatigue identification model.

#### 4.2.1. Feature Selection of Fatigue State

In this paper, the Friedman test is used to analyze 21 ECG characteristic indexes of non-fatigue, mild fatigue, and fatigue state in the training set. These indexes include 8 time domains, 8 frequency domains, and 5 non-linear ones. From these 21 indexes, the indexes with significant differences regarding fatigue state were selected by the Friedman test.

(1) Time domain characteristics

The time domain characteristic indexes selected by the Friedman test are AVNN, AVHR, RMSSD, and PNN50, and the results are listed in Table 7. The asymptotic significance of these indexes is 0.000 < 0.05, which shows that these four time domain indexes have significant differences in non-fatigue, mild fatigue, and fatigue states.

**Figure 10:**
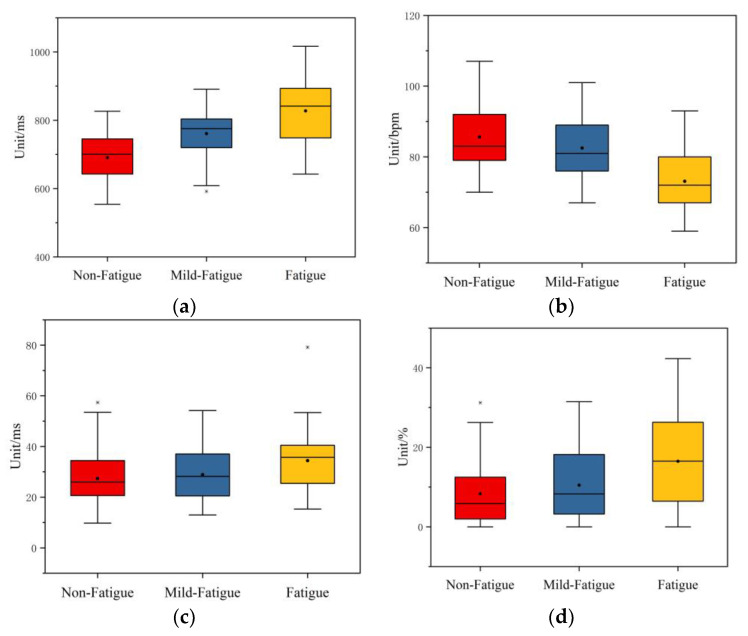
The boxplot of the time domain indexes after feature selection. (**a**) The boxplot of AVNN. (**b**) The boxplot of AVHR. (**c**) The boxplot of RMSSD. (**d**) The boxplot of PNN50.

The boxplot of the time domain indexes selected by the Friedman test is shown in Figure 10. The maximum and minimum in the boxplot are the maximal non-abnormal value and the minimal non-abnormal value, respectively. The number of outliers is extremely small, and individual outliers do not affect the results. Therefore, detailed analysis of outliers was not performed [35,47]. The maximum, minimum, median, average, and upper/lower quartiles of AVNN increased by the increase in degree of fatigue. The maximum, minimum, median, average, and upper/lower quartiles of AVHR decreased with the increase in degree of fatigue. The minimum, median, average, and upper/lower quartile of RMSSD increase with the increase in degree of fatigue. The maximum, median, average, and upper/lower quartile of PNN50 increased with the increase in degree of fatigue. It can be seen that AVNN, RMSSD, and PNN50 have an obvious upward trend, while AVHR has a significant downward trend with the increase in degree of fatigue.

(2) Frequency domain characteristics

After the Friedman test, the selected frequency characteristic indexes are SD1, A++, and B++, and the results are exhibited in Table 8. The asymptotic significances of these indexes are 0.000, 0.001, and 0.007, respectively, which are less than 0.05. This shows that these time frequency indexes have significant differences in non-fatigue, mild fatigue, and fatigue states.

**Figure 11:**
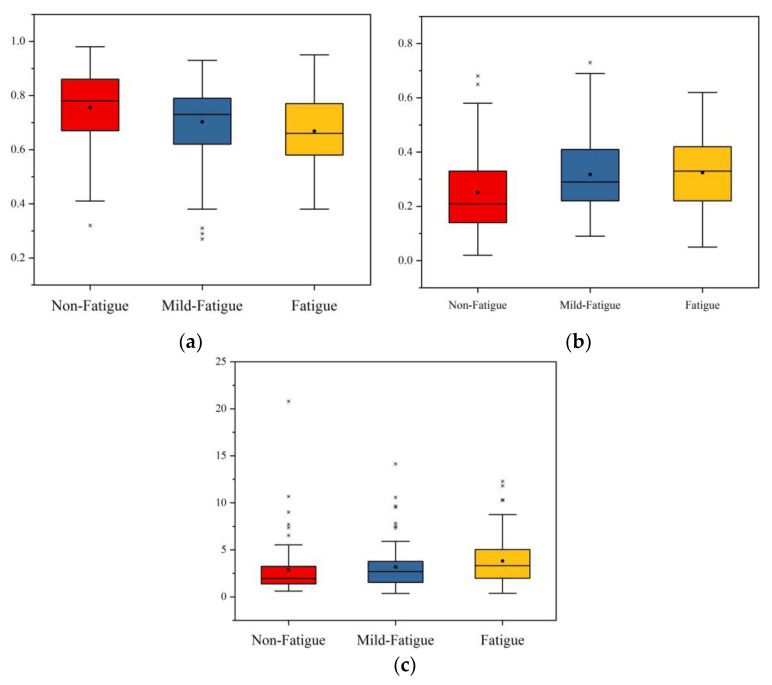
The boxplot of the frequency domain indicator after feature selection. (**a**) The boxplot of LFnorm. (**b**) The boxplot of HFnorm. (**c**) The boxplot of LF/HF.

The boxplot of the frequency domain indexes after feature selection is found in Figure 11. The median, average, and upper/lower quartile of LFnorm decrease with the increase in degree of fatigue. As fatigue increases, the median, average, and upper/lower quartile of HFnorm is increased. The maximum, median, average, and upper/lower quartile of LF/HF decrease with the increase in degree of fatigue. Therefore, as fatigue increases, LFnorm and LF/HF have a significant downward trend, while LFnorm has a significant upward trend.

(3) Non-linear characteristics

After the Friedman test, the selected nonlinear characteristic indexes are SD1, A++, and B++, and the results are given in Table 9. The asymptotic significances of these indexes are 0.001, 0.000, and 0.001, respectively, which are less than 0.05. This indicates that these three time domain indexes have significant differences in non-fatigue, mild fatigue, and fatigue states.

**Figure 12:**
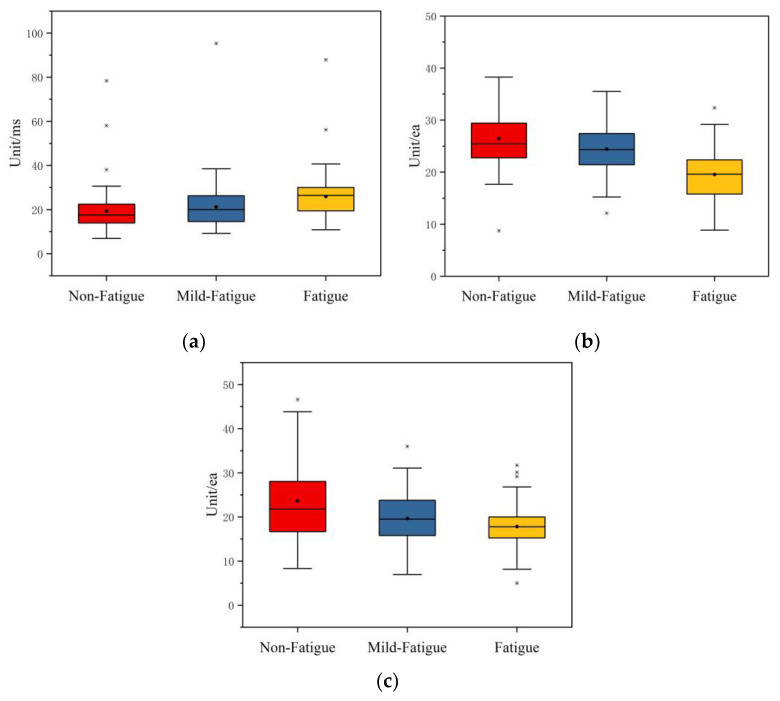
The boxplot of the non-linear indexes after feature selection. (**a**) The boxplot of SD1. (**b**) The boxplot of A++. (**c**) The boxplot of B++.

The boxplot of the non-linear indexes after feature selection is shown in Figure 12. The maximum, minimum, median, average, and upper/lower quartile of SD1 increase with the increase in fatigue. The maximum, minimum, median, average, and upper/lower quartile of A++ decrease with the increase in fatigue. As fatigue increases, the maximum, median, average, and upper quartiles of B++ are decreased. It can be seen that with the increase in fatigue, SD1 has a significant upward trend, while A++ and B++ have a significant downward trend.

In this paper, the Friedman test is adopted to select the features in time domain, frequency domain, and non-linear indexes. The feature selection results show that AVNN, AVHR, RMSSD, PNN50, LFnorm, HFnorm, LF/HF, SD1, A++, and B++ are statistically different in non-fatigue, mild fatigue, and fatigue states.

#### 4.2.2. Feature Extraction of Fatigue State

**Figure 13:**
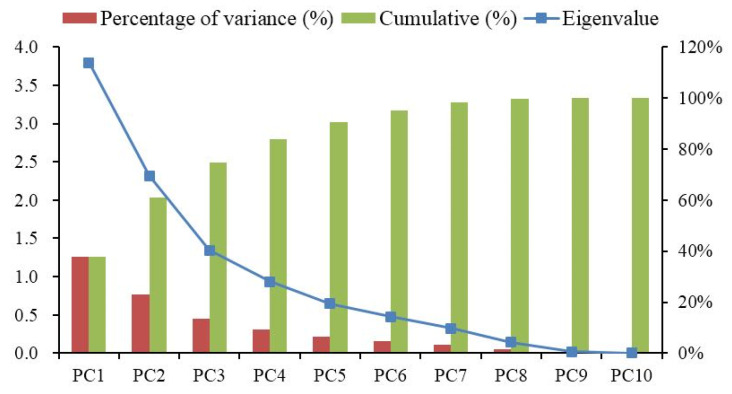
Principal component analysis results.

PCA is adopted to further eliminate the mutual effect among feature extraction. The results based on PCA are depicted in Figure 13. The variance contribution rates of the first five principal components (PC1, PC2, PC3, PC4, and PC5) are 37.94%, 23.17%, 13.47%, 9.36%, and 6.46%, respectively. The cumulative contribution rate of the first five principal components is 90.4%. Accordingly, PC1, PC2, PC3, PC4, and PC5 are used as principal components. The factor loading matrix of five principal components are described in Table 10. As can be seen from Table 10, the main components of PC1 include AVNN and AVHR. The main components of PC2 are LFnorm, HFnorm, and LF/HF. The main components of PC3 are RMSSD, SD1, and B++. The main components of PC4 include PNN50 and A++. Among them, A++ plays a decisive role in PC4, which is 0.98. The main components of PC5 are PNN50 and B++. According to the feature extraction results by principal component analysis, PC1, PC2, PC3, PC4, and PC5 are selected as new feature indexes for model training.

### 4.3. Identification of Fatigue State

Taking into account the classification accuracy and recognition speed of the network, the pilots’ fatigue state identification model is established based on the training set. The test set is used to verify the model. Moreover, it is compared with the traditional classification methods, such as SVM and BPNN.

#### 4.3.1. Establishment of LVQ Model

Based on the results of feature extraction from the training set, there are five feature indexes, namely, principal components PC1, PC2, PC3, PC4, and PC5. These five feature indexes are utilized as inputs for training, so the number of neurons in the input layer is five. The number of neurons in the output layer is three, corresponding to the three states of non-fatigue, mild fatigue, and fatigue. In the model training process, the number of iteration steps is 1000, the learning rate is 0.01, and the target error is 0.1.

**Figure 14:**
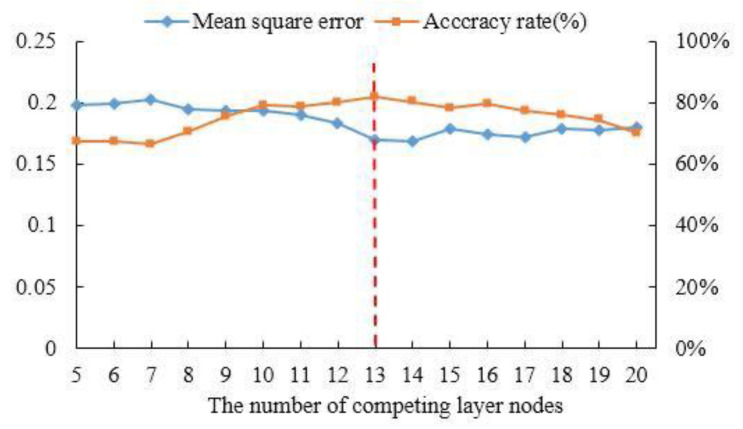
The accuracy rate and MSE of different numbers of neurons.

The number of neurons in the competitive layers has a great effect on the performance of the LVQ model. An excessive number of neurons in competitive layers will increase the complexity of the model and increase the training time. Too few neurons in the competition layer will make the model too simple and poor fitting. The mean square error (MSE) is chosen as the loss function. The smaller the value of MSE, the closer the model output distribution and the sample label distribution are. Here, K-fold cross validation (K = 10) is adopted to determine the best number of neurons in the competitive layer through a large number of experiments. The accuracy rate and MSE of pilots’ fatigue state identification based on the LVQ model are illustrated in Figure 14. When the number of neurons in the competitive layer is 13, the identification accuracy rate is the highest, and the MSE is relatively small, so the number of neurons in the competitive layer is 13.

#### 4.3.2. Analysis of Identification Results

**Figure 15:**
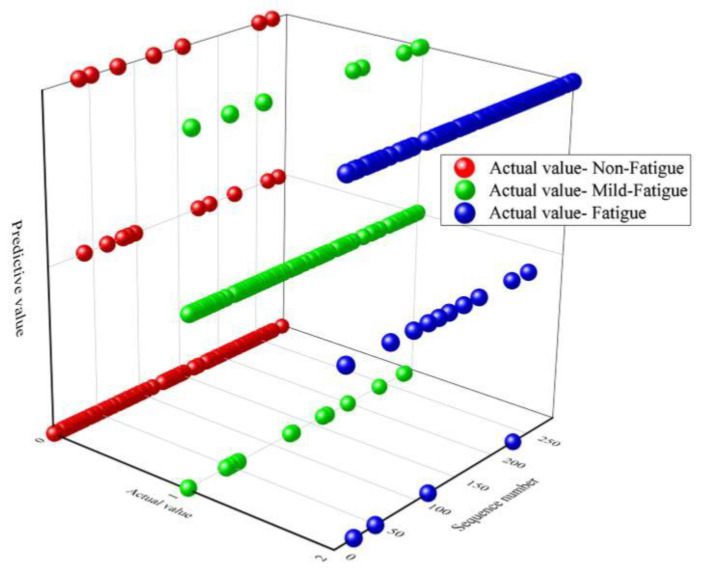
Identification results of the pilots’ fatigue state based on the LVQ model.

Based on the results of feature selection and extraction from the training set, AVNN, AVHR, RMSSD, PNN50, LFnorm, HFnorm, LF/HF, SD1, A++, and B++ were selected from the test set. After feature selection, the first five principal components were extracted from ten indexes in the test set. The first five principal components were input into the LVQ model to test the model. The pilots’ fatigue state identification results in the test set are revealed in Figure 15. In the figure, “0”, “1”, and “2” are, respectively, used to represent the non-fatigue, mild fatigue, and fatigue states. In the 96 test samples with a real label of non-fatigue state, 10 are identified as mild fatigue state, and seven are identified as fatigue state. In the 96 test samples with a real label of mild fatigue state, 13 are identified as non-fatigue state, and eight are identified as fatigue state. In the 96 test samples with a real label of fatigue state, four are identified as non-fatigue state, and 10 are identified as mild fatigue state. The identification accuracy rate of the pilots’ fatigue state is listed in Table 11. The average identification accuracy rate of the LVQ model is 81.94%, which can effectively identify the pilots’ non-fatigue, mild fatigue, and fatigue state.

#### 4.3.3. Model Performance Evaluation

**Figure 16:**
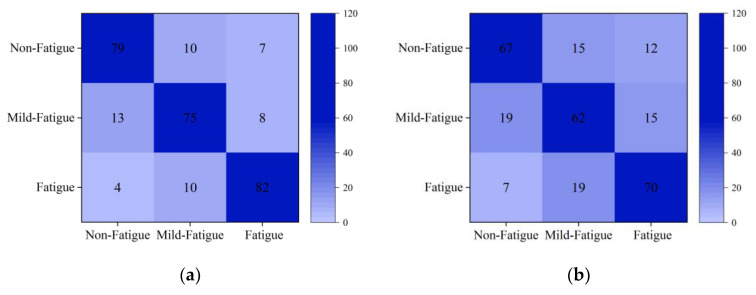

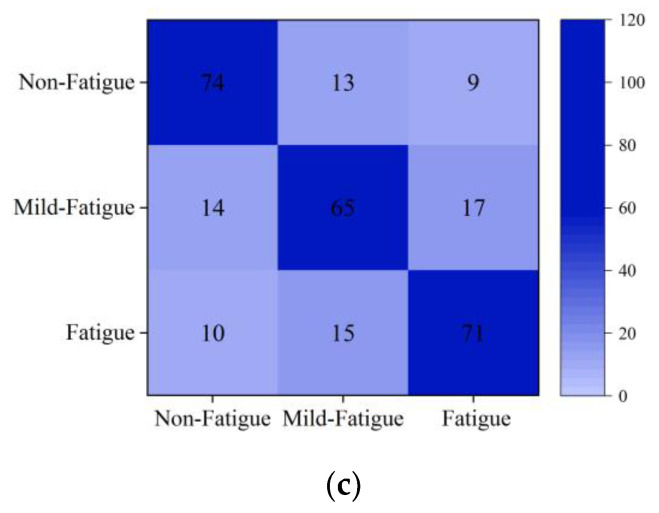
Confusion matrix of the three classification models. (**a**) Confusion matrix of LVQ model. (**b**) Confusion matrix of BPNN model. (**c**) Confusion matrix of SVM model.

In this paper, the identification results of the LVQ model are compared with those of traditional classification models SVM and BPNN to verify the accuracy and effectiveness of the model. The training set is utilized to train SVM and BPNN, respectively. Additionally, the test set is used to test the SVM and BPNN model. The confusion matrixes of the LVQ model, SVM model, and BPNN model are described in Figure 16, and the identification results are exhibited in Table 12. Figure 16 shows that the accuracy rates of the LVQ model in three kinds of fatigue state are 82%, 78%, and 85%, respectively. The accuracy rates of the BPNN model in three kinds of fatigue state are 70%, 65%, and 73%, respectively. The accuracy rates of the SVM model in three kinds of fatigue state are 77%, 68%, and 73%, respectively. The accuracy rates of the LVQ model in three kinds of fatigue state are higher than those of the BPNN model and SVM model. It can be seen from Table 12 that the recognition accuracy of the LVQ model is 12.84% higher than that of the BPNN model and 9.02% higher than that of the SVM model. Therefore, the pilots’ fatigue identification model based on the LVQ model established in this paper has a high identification accuracy.

In order to further evaluate the performance of the classification model, a precision rate, recall value, F1 index, and ROC curve are adopted. Based on the results of the model on the test set, the model evaluation indexes are calculated. The precision rate, recall value and F1 index are calculated by Equations (9)–(11). The precision rate is the proportion of the number of positive samples in the total number of positive samples in the prediction classification, which reflects the accuracy of classification. The recall value is the proportion of the quantity of positive samples in the classification, which reflects the sensitivity of the model. The F1 index is the weighted harmonic average of precision and recall, which reflects the overall index. When the F1 index is high, it indicates that the classification method is more effective. The calculation results of the precision rate, recall value, and F1 index of the LVQ, BPNN, and SVM models are displayed in Table 13. The performance of the LVQ model in the precision rate, recall value, and F1 index is significantly better than that of the BPNN and SVM models. The LVQ model has a higher classification accuracy and model sensitivity than that of the BPNN model and SVM model.

(9) $$Precision=\frac{TP}{TP+FP}\times 100\%$$

(10) $$Recall=\frac{TP}{TP+FN}\times 100\%$$

(11) $$F1-Score=\frac{2\times Precision\times Accuracy}{Precision+Accuracy}\times 100\%$$

where $Accuracy=\frac{TP+TN}{TP+FP+TN+FN}$. *TP* is the abbreviation of True Positive. It represents that the actual results and predicted results are both positive. *FP* is the abbreviation of False Positive. It represents that the actual result is opposite to the predicted result. *TN* is the abbreviation of True Negative. It represents that the actual results and predicted results are both negative. *FN* is the abbreviation of False Negative. It represents that the actual result is opposite to the predicted result.

**Figure 17:**
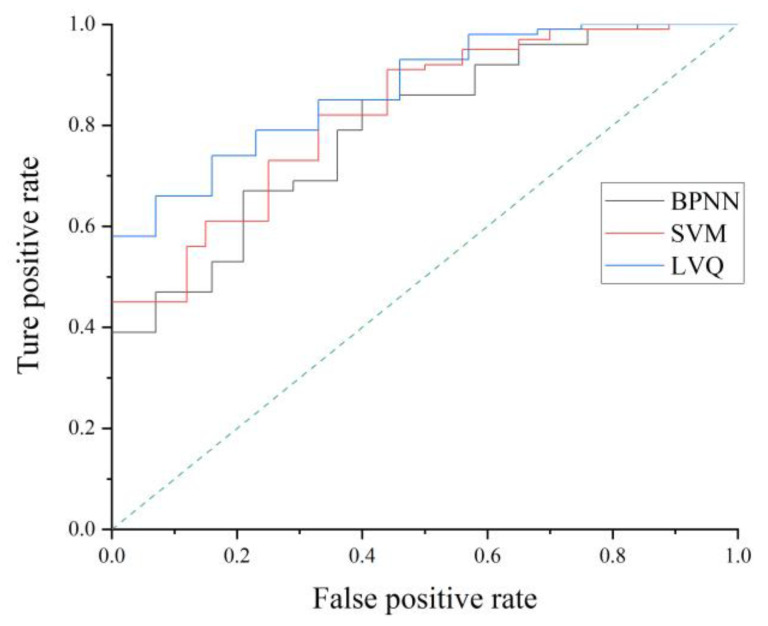
ROC curves of three models.

The ROC curve is a comprehensive indicator, which reflects the continuous variables of sensitivity and specificity. The receiver operating characteristics curves of the LVQ, BPNN, and SVM models are described in Figure 17. Three curves are located at the top left of the 45° diagonal and deviate from the 45° diagonal. Three models have good recognition performance. The curve of the LVQ model is closer to (0,1) point than that of the SVM model and BPNN model, which indicates that the performance of the LVQ model is better.

The precision rate, recall value, F1 index, and ROC curve results of the LVQ model, SVM model, and BPNN model show that the pilots’ fatigue state identification model based on the LVQ model is reasonable, stable, and effective. The pilots’ fatigue state identification model based on LVQ in this paper has high accuracy and reliability.

Based on the above research, in order to build the pilots’ fatigue state identification model, the pilot is selected as the participant, the airfield traffic pattern is the simulated flight task, and the wireless wearable ECG device is the data acquisition device. The simulated flight experiment has less interference in the pilot’s operation, and the cost of data acquisition is low. Hence, the obtained ECG data are of great significance for the study of pilots’ fatigue state identification and the trends of variation in ECG signal indicators under different fatigue states.

In this paper, the indexes that can characterize pilots’ fatigue state are obtained through the Friedman test. The characteristics of each indicator are extracted based on the PCA. Based on the extracted features, the LVQ is adopted to establish pilots’ fatigue state identification model. After model evaluation and verification, the modeling process is scientific and reasonable, which has reference significance for pilots’ fatigue state identification.

Due to the limitation of time and energy, this paper only divides the pilots’ fatigue state into non-fatigue, mild fatigue, and fatigue. In future research, the pilots’ fatigue state could be further refined and identified. This paper only compares the data using the three fatigue states and does not explore the change rule of each index in each fatigue state in detail. Moreover, the change of each indicator in each fatigue state could be explored. Here, the participants selected for the experiment are all male pilots, and female pilots could be recruited for the experiment in future research to expand the research scope. In this paper, 30 pilots were recruited as the participants of the flight simulation experiment. In future studies, the number of participants could be increased, and the reliability could be improved by increasing the sample size.

## 5. Conclusions

In this paper, a wearable wireless ECG device was adopted to obtain pilots’ ECG data in a flight simulation experiment, and 1440 valid samples were selected. Then, the Friedman test was adopted to filter out the characteristic indexes that reflect the pilots’ fatigue state from the time domain, frequency domain, and non-linear characteristics in the training set. AVNN, AVHR, RMSSD, and PNN50 are the time domain indexes; LFnorm, HFnorm, and LF/HF are the frequency domain indexes; and SD1, A++, and B++ are the non-linear indexes. These selected characteristic indexes have statistical differences in non-fatigue, mild fatigue, and fatigue states, which could be used to quantitatively identify the pilots’ fatigue states. This lays a foundation for more scholars to identify pilots’ fatigue state based on ECG signals and study pilots’ fatigue mechanisms. In order to reduce the correlation between feature indexes and improve the speed and accuracy of training, PCA was utilized to extract features from the selected feature indexes. Additionally, five principal components were extracted from 10 feature indexes to establish a feature parameter set. The characteristic parameter set was used as the input of the LVQ model to train the pilots’ fatigue state identification model. The recognition accuracy of the LVQ model was 81.94%, which is 12.84% and 9.02% higher than that of the BPNN and SVM models, respectively. Therefore, the pilots’ fatigue state identification model based on the LVQ model established in this paper has a high identification accuracy. The present results provide a theoretical basis for reducing flight accidents caused by pilot fatigue. At the same time, the results also provide a practical reference for pilot fatigue risk management and the development of intelligent aircraft autopilot systems.

## Figures and Tables

**Table 1:** ECG signal denoising.

Noise Type	Denoising Method	Notes
White noise	Wavelet threshold denoising	Wavelet threshold denoising method is used to decompose ECG signal and remove white noise and motion artifacts
motion artifacts		
Power line interference	Band Pass filter	Filter out 50 Hz power line interference
EMG interference	Low Pass filter	Filter out high-frequency noise greater than 100 Hz
Baseline drift	High Pass filter	Filter out low-frequency noise below 0.5 Hz

**Table 2:** Time domain, frequency domain and non-linear indexes.

	Indicators	Description	Unit	Symbol
Time Domain	AVNN	Mean R-R interval	ms	T_1_
	AVHR	Average heart rate	bpm	T_2_
	SDNN	The standard deviation of R-R interval index	ms	T_3_
	CV	Coefficient of variation		T_4_
	RMSSD	The root mean square of successive R-R interval	ms	T_5_
	SDSD	Standard deviation of successive R-R interval	ms	T_6_
	PNN50	The percentage of successive normal cardiac inter-beat intervals greater than 50 ms	%	T_7_
	PNN20	The percentage of successive normal cardiac inter-beat intervals greater than 20 ms	%	T_8_
Frequency Domain	LF	Low-frequency power (0.04~0.15 Hz), generally reflects the activity information of sympathetic nerves	ms^2^	F_1_
	LF Percent	The percentage of low-frequency power	%	F_2_
	LFnorm	Normalized low-frequency power, reflects the human body’s dual regulation of sympathetic and parasympathetic nerve activities		F_3_
	HF	High-frequency power (0.15~0.4 Hz), reflects the regulation of vagus nerve activity	ms^2^	F_4_
	HF Percent	The percentage of high-frequency power	%	F_5_
	HFnorm	Normalized high-frequency power, reflects changes in parasympathetic regulation		F_6_
	TP	Total power, reflects total variability of ECG signal	ms^2^	F_7_
	LF/HF	Low-frequency power/high-frequency power, reflects the balance of sympathetic and parasympathetic nervous system		F_8_
Non-Linear Domain	SD1	Major axis of the ellipse of Poincaré plot	ms	N_1_
	SD2	Minor axes of an ellipse of Poincaré plot	ms	N_2_
	S	The area of the fitted ellipse over Poincaré plot	ms^2^	N_3_
	A++	The number of points in the first quadrant in scatter plot	%	N_4_
	B−−	The number of points in the third quadrant in scatter plot	%	N_5_

Notes: Scatter plot is the coordinate point obtained by difference of three consecutive IBI points.

**Table 3:** Basic information of the participants.

Basic Information	Mean and Standard Deviation
Age	23.40 ± 1.60 (year)
Mean time of flight	273.24 ± 22.14 (h)

**Table 4:** Samn–Perelli 7-Level fatigue scale.

Item	Description	Scale
1	Very alert and fully awake	1
2	Very energetic but no longer at one’s peak	2
3	Some vitality	3
4	A little tired and lacking energy	4
5	Moderate fatigue	5
6	Very tired and difficult in concentrating	6
7	Exhausted	7

**Table 5:** Parameters of wireless wearable ECG sensor.

Name	Value	Name	Value
Number of channels	6	Sampling rate	512 Hz
Resolution	≥16 Bit	Common mode rejection ratio	110 dB
Noise	≤1.6 μV (RMS)	Measurement range	−1500 μV~+1500 μV
Basic magnification	500	Wireless transmission frequency	2.4 GHz
Accuracy	0.183 μV	Wireless sensor weight	≤20 g

**Table 6:** Some experimental sample data.

NO. 1	Gender	Male	Age	24	Fatigue State	Non-Fatigue	
T_1_	T_2_	T_3_	T_4_	T_5_	T_6_	T_7_	T_8_
736.04	82	29.26	10.47	19.02	28.77	27.85	69.62
F_1_	F_2_	F_3_	F_4_	F_5_	F_6_	F_7_	F_8_
387.11	43.07	0.7	1045.83	18.41	0.3	5682.27	2.34
N_1_	N_2_	N_3_	N_4_	N_5_			
35.36	103.13	11,456.37	18	21			
NO.2	Gender	Male	Age	24	Fatigue state	Non-fatigue	
T_1_	T_2_	T_3_	T_4_	T_5_	T_6_	T_7_	T_8_
724.56	83	37.29	12.94	19.51	33.99	17.5	66.25
F_1_	F_2_	F_3_	F_4_	F_5_	F_6_	F_7_	F_8_
551.54	42.33	0.7	1261.26	17.74	0.3	7111.41	2.39
N_1_	N_2_	N_3_	N_4_	N_5_			
38.95	126.7	15,503.65	22	18			
⋮	⋮	⋮	⋮	⋮	⋮	⋮	⋮
NO.1440	Gender	Male	Age	24	Fatigue state	Fatigue	
T_1_	T_2_	T_3_	T_4_	T_5_	T_6_	T_7_	T_8_
900.45	67	87.58	6.21	40.1	40.41	20.31	57.81
F_1_	F_2_	F_3_	F_4_	F_5_	F_6_	F_7_	F_8_
2079.52	57.06	0.9	230.82	6.33	0.1	3644.33	9.01
N_1_	N_2_	N_3_	N_4_	N_5_			
28.57	73.74	6618.56	14	15			

**Table 7:** Feature selection results of time domain indexes.

Time Domain Indexes	Fatigue States	Average Value	Standard Deviation	Mean Rank	Chi-Square Value	df	Asymp. Sig.
AVNN	Non-fatigue	698.1821	72.34479	1.49	53.106	2	0.000
	Mild fatigue	740.2675	75.84125	1.91			
	Fatigue	817.9340	92.29630	2.60			
AVHR	Non-fatigue	87.2824	9.33199	2.51	53.042	2	0.000
	Mild fatigue	82.0353	8.54602	2.09			
	Fatigue	74.3765	8.43882	1.41			
RMSSD	Non-fatigue	28.5129	13.26642	1.74	20.988	2	0.000
	Mild fatigue	30.0260	15.26040	1.86			
	Fatigue	36.6806	14.48879	2.40			
PNN50	Non-fatigue	8.8676	8.83414	1.66	32.645	2	0.000
	Mild fatigue	10.3058	8.53710	1.84			
	Fatigue	17.0691	10.98281	2.49			

Notes: The asymptotic significance is lower than 0.05, which indicates that the corresponding samples have significant differences.

**Table 8:** Feature selection results of frequency domain indexes.

Frequency Domain Indexes	Fatigue States	Average Value	Standard Deviation	Mean Rank	Chi-Square Value	df	Sig.
LFnorm	Non-fatigue	0.7448	0.14369	2.34	16.309	2	0.000
	Mild fatigue	0.6822	0.14964	1.92			
	Fatigue	0.6704	0.12799	1.74			
HFnorm	Non-fatigue	0.2653	0.15050	1.65	16.617	2	0.001
	Mild fatigue	0.3221	0.15624	2.12			
	Fatigue	0.3320	0.13046	2.24			
LF/HF	Non-fatigue	3.1445	3.24117	1.81	9.812	2	0.007
	Mild fatigue	3.3964	2.83835	1.92			
	Fatigue	4.1736	3.50645	2.27			

Notes: The asymptotic significance is lower than 0.05, which indicates that the corresponding samples have significant differences.

**Table 9:** Feature selection results of non-linear domain indexes.

Non-Linear Domain Indexes	Fatigue States	Average Value	Standard Deviation	Mean Rank	Chi-Square Value	df	Asymp. Sig.
SD1	Non-fatigue	20.1111	9.25549	1.75	19.694	2	0.001
	Mild fatigue	21.2862	10.89284	1.86			
	Fatigue	26.3768	10.33911	2.39			
A++	Non-fatigue	25.2524	4.81696	2.35	35.671	2	0.000
	Mild fatigue	24.2551	4.76400	2.16			
	Fatigue	19.6632	4.71692	1.48			
B++	Non-fatigue	23.0033	8.76712	2.29	17.435	2	0.001
	Mild fatigue	20.2130	5.50636	2.05			
	Fatigue	17.7637	4.76061	1.66			

Notes: The asymptotic significance is lower than 0.05, which indicates that the corresponding samples have significant differences.

**Table 10:** The factor loading matrix of PCA.

	PC1	PC2	PC3	PC4	PC5
AVNN	0.43754	0.12881	−0.32497	0.0453	0.21814
AVHR	−0.43284	−0.12248	0.3506	−0.07952	−0.14701
RMSSD	0.39995	0.15475	0.44308	0.0385	-0.32202
PNN50	0.38485	0.11403	0.11148	0.12194	0.56775
LFnorm	−0.16641	0.50146	−0.17952	0.05901	0.07257
Hfnorm	0.14774	−0.59501	0.03596	0.0402	0.09042
LF/HF	−0.15979	0.54117	−0.01296	−0.04581	−0.0966
SD1	0.40045	0.15491	0.44214	0.03842	−0.32096
A++	−0.15474	−0.01594	0.04499	0.98063	−0.03578
B++	−0.2319	0.09409	0.57578	−0.07052	0.61457

**Table 11:** Identification accuracy rate based on the LVQ model.

Classification	Non-Fatigue	Mild Fatigue	Fatigue	Average
Accuracy (%)	82.29	78.13	85.42	81.94

**Table 12:** Model identification accuracy.

Model	LVQ	BPNN	SVM
Accuracy (%)	81.94	69.10	72.92

**Table 13:** Performance evaluation results of classification models.

Method	Precision (%)	Recall (%)	F1-Score (%)
LVQ	81.93	81.94	81.93
BPNN	69.60	69.10	69.35
SVM	72.87	72.92	72.89
